# Genome-Wide DNA Methylation Patterns in Persistent Attention-Deficit/Hyperactivity Disorder and in Association With Impulsive and Callous Traits

**DOI:** 10.3389/fgene.2020.00016

**Published:** 2020-01-31

**Authors:** Mandy Meijer, Marieke Klein, Eilis Hannon, Dennis van der Meer, Catharina Hartman, Jaap Oosterlaan, Dirk Heslenfeld, Pieter J. Hoekstra, Jan Buitelaar, Jonathan Mill, Barbara Franke

**Affiliations:** ^1^ Department of Human Genetics, Donders Institute for Brain, Cognition and Behavior, Radboud University Medical Center, Nijmegen, Netherlands; ^2^ Department of Psychiatry, UMC Utrecht Brain Center, University Medical Center Utrecht, Utrecht, Netherlands; ^3^ Medical School, University of Exeter, Exeter, United Kingdom; ^4^ NORMENT, Division of Mental Health and Addiction, Oslo University Hospital & Institute of Clinical Medicine, University of Oslo, Oslo, Norway; ^5^ Faculty of Health, Medicine and Life Sciences, School of Mental Health and Neuroscience, Maastricht University, Maastricht, Netherlands; ^6^ Department of Psychiatry, University Medical Center Groningen, University of Groningen, Groningen, Netherlands; ^7^ Experimental and Clinical Neuropsychology Section, Vrije Universiteit Amsterdam, Amsterdam, Netherlands; ^8^ Emma Neuroscience Group, Department of Pediatrics, Amsterdam Reproduction & Development, Emma Children's Hospital, Amsterdam UMC, University of Amsterdam and Vrije Universiteit Amsterdam, Amsterdam, Netherlands; ^9^ Donders Centre for Cognitive Neuroimaging, Donders Institute for Brain, Cognition and Behavior, Radboud University, Nijmegen, Netherlands; ^10^ Department of Cognitive Neuroscience, Radboud University Medical Centre, Nijmegen, Netherlands; ^11^ Karakter Child and Adolescent Psychiatric University Centre, Nijmegen, Netherlands; ^12^ Department of Psychiatry, Donders Institute for Brain, Cognition and Behavior, Radboud University Medical Center, Nijmegen, Netherlands

**Keywords:** persistent attention-deficit/hyperactivity disorder, remittent attention-deficit/hyperactivity disorder, callous traits, impulsivity, DNA methylation, epigenome-wide association study

## Abstract

Attention-deficit/hyperactivity disorder (ADHD) is a neurodevelopmental disorder that often persists into adulthood. ADHD and related personality traits, such as impulsivity and callousness, are caused by genetic and environmental factors and their interplay. Epigenetic modifications of DNA, including methylation, are thought to mediate between such factors and behavior and may behave as biomarkers for disorders. Here, we set out to study DNA methylation in persistent ADHD and related traits. We performed epigenome-wide association studies (EWASs) on peripheral whole blood from participants in the NeuroIMAGE study (age range 12–23 years). We compared participants with persistent ADHD (n = 35) with healthy controls (n = 19) and with participants with remittent ADHD (n = 19). Additionally, we performed EWASs of impulsive and callous traits derived from the Conners Parent Rating Scale and the Callous-Unemotional Inventory, respectively, across all participants. For every EWAS, the linear regression model analyzed included covariates for age, sex, smoking scores, and surrogate variables reflecting blood cell type composition and genetic background. We observed no epigenome-wide significant differences in single CpG site methylation between participants with persistent ADHD and healthy controls or participants with remittent ADHD. However, epigenome-wide analysis of differentially methylated regions provided significant findings showing that hypermethylated regions in the *APOB* and *LPAR5* genes were associated with ADHD persistence compared to ADHD remittance (p = 1.68 * 10^−24^ and p = 9.06 * 10^−7^, respectively); both genes are involved in cholesterol signaling. Both findings appeared to be linked to genetic variation in cis. We found neither significant epigenome-wide single CpG sites nor regions associated with impulsive and callous traits; the top-hits from these analyses were annotated to genes involved in neurotransmitter release and the regulation of the biological clock. No link to genetic variation was observed for these findings, which thus might reflect environmental influences. In conclusion, in this pilot study with a small sample size, we observed several DNA-methylation–disorder/trait associations of potential significance for ADHD and the related behavioral traits. Although we do not wish to draw conclusions before replication in larger, independent samples, cholesterol signaling and metabolism may be of relevance for the onset and/or persistence of ADHD.

## Introduction

Attention-deficit/hyperactivity disorder (ADHD) is a neurodevelopmental disorder with a prevalence of 5% in children (Polanczyk and Rohde, 2007). Of these children, 65% show persistent symptoms of inattention and/or hyperactivity and impulsivity during adulthood (Faraone et al., 2015), but it remains unknown, why some patients show remittance and others persistence of the disorder. Based on twin studies, the heritability of the disorder has been estimated to be ~74% (Faraone and Larsson, 2019), and a recent genome-wide association study assessed the narrow-sense heritability based on common genetic variants to be ~22% (Demontis et al., 2019). Several studies have also indicated the importance of the environment in the development of ADHD, such as prenatal maternal stress, prenatal exposure to toxins, low birth weight (Sciberras et al., 2017), and postnatal stress (Roskam et al., 2014).

One of the core symptoms of ADHD is an increased level of the behavioral trait impulsivity. Another trait associated with ADHD (Fowler et al., 2009), callousness, is predictive of offensive and aggressive behavior (Watson and Clark, 2019; Docherty et al., 2019). Both traits are normally distributed in the general population (Chamorro et al., 2012; Georgiev et al., 2013), and the extreme ends of their spectra are marked as maladaptive and pathological (McLennan, 2016). Both genes (Brikell et al., 2018; Moore et al., 2019) and the environment (Gescher et al., 2018; Waller et al., 2018) play important roles in the etiology of impulsive and callous traits.

Environmental adversities may act on behavior through epigenetic mechanisms (Kubota et al., 2012). Epigenetic mechanisms control gene expression downstream of both environmental and genetic (risk) factors; for example, the methylation of DNA at CpG sites can change in response to an environmental stimulus (Kubota et al., 2012), which can then influence the expression levels of genes, and some differentially methylated genes linked to impulsive and callous behaviors and related disorders like ADHD have recently been described (Waltes et al., 2016; Hamza et al., 2017; Gescher et al., 2018). Since environmental factors can play a role in the onset of ADHD and associated symptoms (Thapar and Cooper, 2016), it is to be expected that DNA methylation differs between healthy controls and people with ADHD (symptoms), which might influence the transcription of genes regulating behavior, as summarized by Palumbo and coworkers for aggressive behavior (Palumbo et al., 2018).

Targeted DNA methylation studies, mostly performed in children, reported on genes in the dopaminergic, adrenergic, and serotonergic system associated with ADHD (Hamza et al., 2017; Dall'Aglio et al., 2018). Only few epigenome-wide association studies (EWAS) of ADHD and ADHD symptoms in the general population have been performed. Such studies were performed in children with and without ADHD (age 7–12 years) (Wilmot et al., 2016), for symptom trajectories in a general population cohort (at birth, age 7, and 7–15 years) (Walton et al., 2017), as well as for an adult cohort of individuals from the general population (age 18–38 years) (van Dongen et al., 2019). Given the fact that a percentage of children with ADHD show remission of the disorder (Biederman et al., 2010), it may be of relevance to study DNA methylation patterns related to the onset of ADHD as well as with its persistence. Up to now, no EWAS investigated the clinical persistence of ADHD into adulthood, representing a lack of hypothesis-generating studies of potential epigenetics-related molecular mechanisms of persistence/remittance of ADHD. Similar to the situation for ADHD, only few previous EWASs investigated the association of DNA methylation and impulsive and aggressive traits, and those involved general population samples, only (van Dongen et al., 2015; Cecil et al., 2018; Montalvo-Ortiz et al., 2018).

In the current pilot study, we aimed to identify methylation differences linked to ADHD persistence, impulsivity, and callousness in the NeuroIMAGE cohort (von Rhein et al., 2015). We hypothesized to find DNA methylation differences between healthy controls and participants with persistent ADHD, whereas participants with remittent ADHD are expected to form an intermediate group. To our knowledge, this is the first study investigating the epigenome of participants with persistent and remittent ADHD. The two behavioral traits we studied are known to be present as continuous traits in the general population and are closely linked to ADHD, but had not directly been selected for in participants of the NeuroIMAGE cohort, enabling us to perform quantitative trait analysis in the mixed population of healthy and affected individuals in the NeuroIMAGE cohort.

## Methods

### Cohort

A subset of 72 independent individuals (persistent ADHD n = 35, remittent ADHD n = 18, healthy control n = 19) were selected based on ADHD symptoms (i.e. sampling groups to cover the extremes of the distribution) from the longitudinal NeuroIMAGE cohort (official cohort n = 751 children with ADHD and n = 318 healthy controls) (Cecil et al., 2018). Participants with remittent ADHD were the scarest group among the three and those were selected first. We included all remitters with complete data availability and DNA isolation from blood. However, inclusion was restricted to one remitter per family. We then matched participants with persistent ADHD and healthy controls to the remitters in terms of age, sex, IQ, and perceived stress using the R package MatchIt v2.4 until reaching the number of 72, excluding family members. Inclusion criteria were European Caucasian descent, IQ > 70, and absence of a diagnosis of autism, epilepsy, general learning difficulties, and known genetic disorders. Participants with a current major depression were excluded. Patients and controls were recruited between 2003 and 2006 (**Supplementary Table 1**). In 2008–2009, participants were re-assessed by telephone interviews, with the same diagnostic criteria where similar loss-of-follow up rates were observed for control (20%) and ADHD (21%) families. Between 2009 and 2012, participants were re-assessed again. During this third assessment, the blood samples for our epigenetics study were taken from the participants. All participants gave written informed consent, and the study was approved by the regional ethics committee (Centrale Commissie Mensgebonden Onderzoek: CMO Regio Arnhem Nijmegen; 2008/163; ABR: NL23894.091.08).

### Assessment of ADHD, Impulsivity, and Callous Traits

ADHD diagnosis was made in accordance with the DSM-IV (Association, 2000) based on the Dutch version of the Parental Account of Children's symptoms (PACS) at baseline (Taylor, 1991) and based on the Schedule for Affective Disorders and Schizophrenia for children (K-SADS) during the first and second follow up, administered to both parents and children to combine information for diagnosis from multiple sources (Kaufman et al., 1997). Participants who were diagnosed with ADHD had ≥6 hyperactive/impulsive and/or inattentive symptoms, met the DSM-IV criteria for pervasiveness and impairment (measures derived from the K-SADS), and showed an age of onset before 12 years. The presence of any other psychiatric disorder was assessed with a procedure similar to the ADHD interview (combination of K-SADS and CPRS).

The scores derived from the PACS and K-SADS were combined with the Conners Teacher Rating Scale: Long version for children <18 years (Conners et al., 1998a) and the Conners Adult ADHD Rating Scale-Self Report: Long version for participants ≥18 years (Conners et al., 1997), depending on the age of the participant (cut-off 18 years). Participants originally diagnosed with ADHD, but not meeting the ADHD diagnostic criteria at follow-up anymore, were classified as remitters (Francx et al., 2015). We did not include participants in our analysis that developed late-onset ADHD. All questionnaires were administered by well-trained research-assistants. Additional information about the diagnostic criteria can be found in the publication of von Rhein and coworker (von Rhein et al., 2015).

To measure impulsivity, the raw scores of the Global restless–impulsive scale I of the Conners Parent Rating Scale (CPRS) were used (Conners et al., 1998b). In total, seven items were scored with either 0 or 1 point: restless or overactive; irritable or impulsive; does not finish the things he/she starts with; has sloppy handwriting; cannot sit still or is wobbling nervously; disturbs other children; and wants desires to be met immediately. Total scores of the Global restless–impulsive scale I of the CPRS can be found in **Table 1** and **Supplementary Figure 1**.

**Table 1:** Characteristics of samples included in the NeuroIMAGE DNA methylation study.

	Healthy control	Remittent ADHD	Persistent ADHD	Total/average	P-value (one-way ANOVA)
**N**	19	18	35	71	
**% Male**	68	67	77	73	0.6683
**Age** **Mean (SD)**	20.12 (2.83)	21.69 (3.38)	20.93 (2.64)	20.91 (2.90)	0.2622
**IQ** **Mean (SD)**	113.83 (10.35)	101.17 (16.11)	96.06 (17.56)	101.94 (16.95)	0.0003* control differs from remittent (4.22) and persistent (6.06)
**DNA methylation smoking score**	5.39 (2.01)	5.82 (2.79)	6.54 (2.64)	6.05 (2.56)	0.2635
**Impulsivity**	1.294 (1.49)	7.611 (5.94)	9.914 (5.43)	7.229 (6.02)	<0.0001* control differs from remittent (5.34) and persistent (8.34)
**Callous**	7.00 (2.22)	9.389 (4.70)	11.086 (5.31)	9.6 (4.84)	0.0114* control differs from remittent (2.22) and persistent (4.36)

*Significant p-value (<0.05).

In the p-value (obtained with one-way-ANOVA) column, number between brackets reflect the Tukey's multiple comparison test q-value for the mentioned groups.

ADHD, attention-deficit/hyperactivity disorder; ANOVA, analysis of variance.

To measure callous traits, participants completed the Inventory of Callous-Unemotional Traits (Kimonis et al., 2008). We created a combined score for callousness by selecting the following nine items reflecting callous behavior: What I think is right and wrong is different from what other people think; I do not care whom I hurt to get what I want; I feel bad or guilty when I do something wrong*; I am concerned about the feelings of others*; I apologize to persons I hurt*; I try not to hurt others' feelings*; I do not feel remorseful when I do something wrong; The feelings of others are unimportant to me; and I do things to make others feel good*. Questions with an * indicate inverse questions, meaning that scores were inverted to calculate callous scores. Questions were scored on a scale of 0–3 (0 = not at all true, 1 = somewhat true, 2 = very true, 3 = definitely true). The final score for a subject could range between 0 and 27. The combined score we created reflects a continuous callous trait, which showed a wider distribution in the selected population than the unemotional component of the behavior, as it also includes offensive behavior.

### DNA Methylation Profiling

DNA was isolated from peripheral blood at the department of Human Genetics of the Radboud University Medical Center in Nijmegen using standard protocols, and an epigenome-wide analysis was performed with the Infinium^®^ MethylationEPIC BeadChip (Illumina, San Diego, USA) by Life & Brain GmbH, Bonn, Germany. The readEPIC() function of the wateRmelon package was used to import methylation data and to compare intensities of methylated (M) and unmethylated (U) DNA (Pidsley et al., 2013). Bisulphite conversion rates were calculated, with samples showing rates < 0.8 excluded. Correlation between 59 common SNPs was used to identify duplicate samples. No probes were excluded based on bisulfite conversion rates or sample duplicates. All probes with bead count <3 in >5% of the samples (n = 9,735) or >1% of samples with a detection p-value >0.05 (n = 4,126) were removed. Beta-values (methylated/unmethylated) were calculated for all probes and were matched to the probe annotation of the EPIC array v1.0 B4 (Illumina Inc., 2017, San Diego, USA) to be used for subsequent analysis. Probes known to cross-hybridize with each other were removed, as were CpG sites with a minor allele frequency <0.05, to be sure effects are because of differentially methylation rather than genomic variation at a CpG site (McCartney et al., 2016). Probes on the X-chromosome that passed quality control were used to verify sex in all participants, by multidimensional scaling (MDS, n components = 2). With the DNA Methylation Age Calculator (https://dnamage.genetics.ucla.edu/home) the age of the subjects at the time of sampling was calculated (Horvath, 2013). Of the 866,554 probes on the array, 853,539 CpG sites passed all QC steps.

### Assessment of Smoking as Covariate

Since smoking is known to affect DNA methylation (Zeilinger et al., 2013), and participants with ADHD indicated to be smokers more often than healthy controls, we corrected for smoking in our model. A smoking score was calculated per individual, based on 177 CpG sites currently known to be differentially methylated by smoking (Zeilinger et al., 2013; Li et al., 2018). The average methylation effect of these CpG sites was calculated based on a discovery and replication cohort as previously described by Elliot and co-workers (Elliott et al., 2014). These smoking scores were used as covariate in the statistical model to account for an individual's smoking status instead of the self-reported smoking status, since this score incorporates past smoking, which was not available at sufficient depth from questionnaires.

Surrogate variable (SV) analysis is a method to capture heterogeneity across the data not caused by the variables of interest and is sensitive enough to detect possible differences of blood cell composition (SV 1 showed a Pearson's correlation = 0.9 with the most abundant blood cell type), ethnical descent, and chip position (Leek and Storey, 2007; Kaushal et al., 2017). SVs were created *via* the “sva” package in R (Leek et al., 2012), and all 10 SVs from the model were included as covariates in the statistical model.

### Statistical Analysis

All analyses were performed in R 3.4.4. Differences in demographics were tested with a one-way-ANOVA. Methylation differences at the single CpG site level were analyzed by means of a linear model with sex, age, smoking score, and 10 SVs as covariates, since sex, age, and smoking are known to affect DNA methylation (Zeilinger et al., 2013; van Dongen et al., 2016). We did not include stimulant medication as a covariate, since there is no evidence that it affects DNA methylation (Wilmot et al., 2016). To prevent overcorrection of the model by including additional covariates of unknown relevance, we included the 10 SVs. We ran the following comparisons: (1) healthy controls (n = 19) and participants with persistent ADHD (n = 35), (2) participants with remittent ADHD (n = 18) and with persistent ADHD. Two additional EWASs were performed for impulsive and callous traits as continuous measures. The study-wide epigenome-wide significant p-value threshold was set at p < 9 * 10^−8^ (Mansell et al., 2019). However, given the exploratory character of our study, we also considered nominally significant results with p < 5 * 10^−5^, unadjusted for the number of EWASs performed. Inflation factors for the regression model were calculated as the square root of the expected median of a chi-squared statistics distribution divided by the median of the chi-statistics with one degree of freedom.

The “Comb-p” Python module was used to identify differentially methylated regions (DMR) in all analyses (Pedersen et al., 2012). A region was considered to be a DMR if at least two methylation sites were differentially methylated within a 500 bp window (Šidák-corrected p-value < 0.05) (Crawford et al., 2018).

### Biological Interpretation of Top-Findings

To assess whether the observed levels of CpG methylation depend on genetic variation, methylation quantitative trait loci (meQTL) were assessed for all nominally significant CpG sites from the former analyses. Databases on meQTLs in peripheral blood during adolescence (Gaunt et al., 2016) and in the dorsal lateral prefrontal cortex in adults (Ng et al., 2017) were used. A SNP with <1 Mb distance to the corresponding CpG site was considered to be a trans-meQTL. In addition, expression quantitative trait methylations (eQTM) were assessed *via* the genome browser of iMETHYL, based on experiments in neutrophils isolated from 100 healthy Japanese subjects (Hachiya et al., 2017).

## Results

The characteristics of the study sample are displayed in **Table 1**. There were no significant differences between healthy controls (n = 19) and participants with remittent (n = 18) and with persistent ADHD (n = 35) for age, sex, and smoking score (**Table 1**), for which the samples had been matched. Healthy controls showed significantly higher IQ than participants with remittent and persistent ADHD (p = 0.0003, Tukey's multiple comparison test q = 4.22 and 6.06, respectively). Impulsivity (p < 0.0001, Tukey's multiple comparison test q = 5.34 and 8.34) and callousness (p = 0.0114, Tukey's multiple comparison test q = 2.22 and 4.36) scores also differed between participants with ADHD and controls. Distributions of impulsive and callous traits overlapped among the different groups, showing a wide spread (**Supplementary Figure 1**). The continuous traits did not correlate with each other (Pearson's r = 0.215, p = 0.073), indicating that the analysis on impulsivity and callousness were supplementary to each other.

### Robust Epigenetic Signatures of Age and Tobacco Use Validate DNA Methylation Data

To verify the robustness of our DNA methylation signal, we aimed to validate reported age by measuring DNA methylation age. The average chronological age predicted from methylation data was 14.78 years (SD = 3.43), and this was strongly correlated with actual age (r = 0.650, p = 6.5 * 10^−10^) (**Supplementary Figure 2A**). Individuals with the highest deviation from reported age were participants with ADHD and smokers. Testing markers of smoking exposure during adulthood, we identified a significant association between the methylation-derived smoking scores and self-reported smoking behavior, with current smokers having higher smoking scores compared to non-smokers (p = 2.38 * 10^−8^, **Supplementary Figure 2B**).

### DNA Methylation Profiles of Healthy Controls and Participants With Persistent and Remittent ADHD Differ

**Figure 1:**
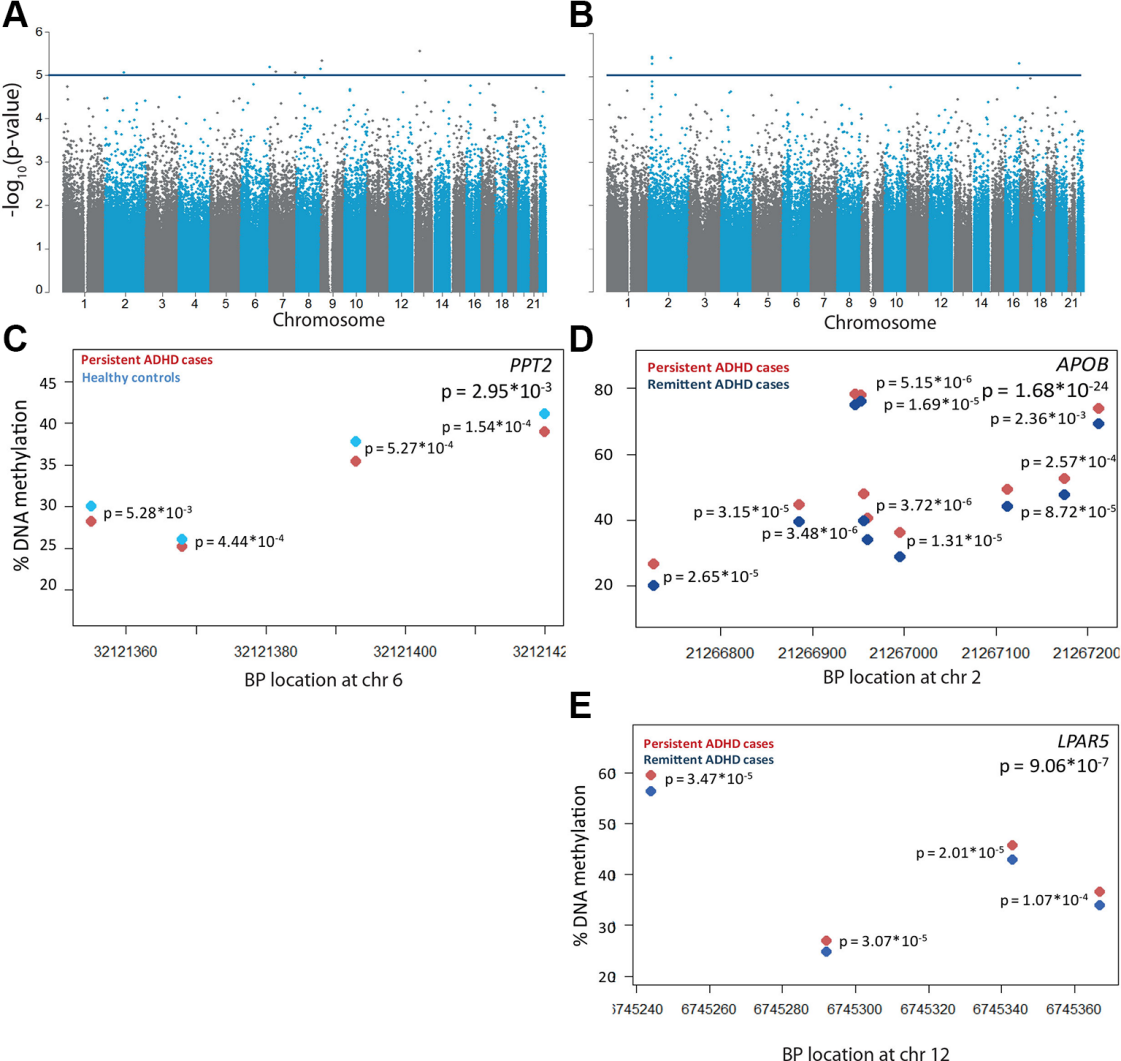
Differential DNA methylation in persistent attention-deficit/hyperactivity disorder (ADHD). −log_10_(p-values) of the methylation sites are plotted along their chromosomal position for **(A)** n = 35 participants with persistent ADHD and n = 19 healthy controls and **(B)** n = 35 participants with persistent and n = 18 participants with remittent ADHD. The horizontal blue line indicates the nominally significance threshold of −log_10_(5). One differentially methylated region was found in **(C)** *PPT2* for participants with persistent ADHD (red) compared to healthy controls (light blue). Differentially methylated regions were found in **(D)** *APOB* and **(E)** *LPAR5* for participants with persistent compared to remittent ADHD (dark blue). Each circle represents a CpG site, of which DNA methylation levels are plotted for the corresponding chromosomal location. Individual p-values per CpG sites are located next to the site, and a combined p-value is given in the upper right corner.

To assess DNA methylation profiles associated with persistent ADHD, we compared methylation levels of participants with persistent ADHD with those of healthy controls, estimated by averaging across all probes on the array included in our analyses. The inflation factor of 0.996 showed that no inflation was present in the model. Here, no global differences in DNA methylation were found between participants with persistent ADHD (mean proportion of methylated CpG sites = 0.616, SD = 0.0009) and healthy controls (mean proportion of methylated CpG sites = 0.615, SD = 0.001, p = 0.2246). While no individual probes passed the experiment-wide significance threshold, seven CpG sites showed differential methylation at our more lenient, suggestive significance threshold of p < 5 * 10^−5^ (**Figure 1A**, **Supplementary Table 2**, **Supplementary Figure 3A**). In the comparison between persistent and remittent ADHD, global differences in DNA methylation did reach significance (mean proportion of methylated CpG sites persisters = 0.616, SD persisters = 0.0009; mean proportion of methylated CpG sites remitters = 0.615, SD remitters = 0.0040; t-test p = 0.00115). Comparing individual DNA methylation differences between participants with persistent and remittent ADHD, we did not identify any differentially methylated probes passing the experiment-wide significance threshold. The inflation factor of 0.969 showed that no inflation-depth explanatory analysis on nominally significn was present in the model. Five CpG sites showed differential methylation at our more lenient, suggestive significance threshold (p < 5 * 10^−5^) (**Figure 1B**, **Supplementary Table 2**, **Supplementary Figure 3B**). In-depth explanatory analysis on nominally significant sites can be found in the **Supplementary Text** and corresponding tables.

In the regional analysis, we identified a small DMR in the *PPT2* gene (65 bp containing four probes) to be significantly hypomethylated in participants with persistent ADHD compared to healthy controls (Šidák-corrected p-value = 0.030, **Figure 1C**). Two additional significant DMRs were found to be associated with ADHD persistence in the comparison between participants with persistent and remittent ADHD after correction for multiple testing. Both DMRs were hypermethylated in the participants with persistent ADHD (**Figures 1D, E**); one was located within 200 bp of the transcription start site of the *APOB* gene, spanning 485 bp, consisting of 10 measured CpGs (Šidák-corrected p-value = 1.66 * 10^−24^, **Figure 1D**), and one was present in the *LPAR5* gene, spanning 123 bp and containing four probes (Šidák-corrected p-value = 9.06 * 10^−7^, **Figure 1E**). Probes in the *APOB* DMR had also been found nominally associated in the single-probe analysis (**Supplementary Table 2**).

### Differential Methylation Observations in Persistent ADHD Might Be Genomic Effects

To assess whether the differential methylation observed in persistent ADHD might be dependent on genetic variation, meQTLs were assessed. There were 3,460 SNP-meQTL pairs for the *APOB* DMR. Forty-seven SNPs were meQTLs for the third CpG site in the *LPAR5* DMR (**Supplementary Table 3**). This suggests that the differences we observed between participants with persistent and remittent ADHD might be genomic effects. Moreover, 8.1% of the measured meQTL SNPs—more than one would expect by chance (X = 5.008, p = 0.025)—were associated (unadjusted p-value < 0.05) with adult, persistent ADHD in a recent GWAS meta-analysis (Rovira et al., 2019), but none of the findings survived correction for multiple testing.

### DNA Methylation Profiles Associated With Impulsive and Callous Traits

**Figure 2:**
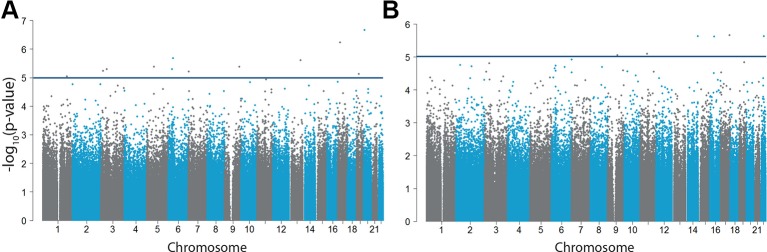
Differential DNA methylation in impulsive and callous traits. −log_10_(p-values) of the methylation sites for **(A)** impulsive traits measured by the CPRS (n = 70) and **(B)** callousness (n = 71). The horizontal blue line indicates our more lenient suggestive significance threshold of −log_10_(5).

No epigenome-wide significant CpG sites correlated with impulsive traits (analyzed in n = 70) (**Figure 2A**, **Supplementary Table 4**) or callousness (**Figure 2B**, **Supplementary Table 4**). For both analyses, no inflation was present showed by an inflation factor of 0.998 and 0.980, respectively. Regional analyses also did not reveal DMRs associated with either impulsive or callous traits.

## Discussion

In the current study, we aimed to 1) identify differences in peripheral blood DNA methylation profiles linked to the persistence of ADHD, and 2) find DNA methylation levels correlated with impulsive and callous traits. To our knowledge, this is the first study in which differences in DNA methylation levels in participants with persistent and remittent ADHD were investigated. This study showed global DNA methylation differences between participants with persistent ADHD compared to participants with remittent ADHD. Two DMRs, in *APOB* and *LPAR5*, were found significantly differentially methylated between participants with remittent and persistent ADHD. Moreover, several CpG sites were differentially methylated between participants with persistent ADHD and healthy controls, and participants with persistent and remittent ADHD based on a more lenient, suggestive significance level of p < 5 * 10^−5^. Lastly, we showed that impulsive and callous traits were associated with 18 differentially methylated CpG sites at this lenient threshold.

### Global DNA Methylation Differences Between Groups

We found that global methylation differences were lower in participants with persistent ADHD than in healthy controls. It is known that DNA methylation gradually decreases during aging (Horvath and Raj, 2018), and it has been shown that DNA methylation based on aging can be accelerated in patients with psychiatric disorders (Han et al., 2018; Okazaki et al., 2019) and in smokers (Zeilinger et al., 2013). This is consistent with our finding that individuals with the largest deviation between actual and predicted age based on DNA methylation (i.e. higher predicted age) were the individuals with ADHD reporting to be smokers.

### Blood DNA Methylation Associated With ADHD Persistence Correlated With Brain DNA Methylation

We expected to find biomarkers of ADHD persistence and associated traits in the DNA isolated from peripheral blood. Such biomarkers do not necessarily reflect brain DNA methylation status, and we have no information on any causal relationships with disease. Some of our findings did show a relatively high correlation with methylation levels in different brain regions. The lack of blood–brain correlation in methylation observed for many of our findings is not necessarily due to the limited sample size of our study. A recent methylome-wide association study for schizophrenia (N = 1448) found that most of their top-findings were also specific for blood, but that half of their sites were located in genes that were enriched in brain-specific GO-terms (Chan et al., 2019). These findings suggest that the DNA methylation markers we identified in blood could also be relevant for brain-related processes and behaviors.

### Comparison With DNA Methylation Sites in Previous (Epi)Genetic Studies

Previous studies investigating the epigenome in ADHD in a candidate gene-based manner have reported differential methylation in the serotonin and dopamine system (Hamza et al., 2017), which had earlier been implicated in ADHD through candidate gene-based genetic studies (Faraone and Larsson, 2019). We did not find any genes related to the dopamine and serotonin systems among our most strongly associated DNA methylation sites for persistent ADHD. While this does not invalidate either the findings from the epigenetic candidate-gene studies nor ours, it may suggest that epigenetic effects in these systems may not be those with the strongest effect sizes. Our observed differential DNA methylation sites also did not overlap with any of the few published EWASs for ADHD, ADHD symptoms, or aggression (Wilmot et al., 2016; Walton et al., 2017; van Dongen et al., 2019). The lack of reproducibility could be explained by our low sample size, the sample populations used (clinical, general population, or mixed cohorts), and the different arrays used for DNA methylation quantification (450K or 850K array).

Based on genetic, rather than epigenetic studies, we did find support for the observed differential methylation sites associated with ADHD. Our most significant finding hints toward fatty acid metabolism in persistent ADHD, and genes involved in fatty acid metabolism and fatty acid oxidation pathways have also been indicated to be differentially methylated in participants with ADHD compared to healthy controls (Wilmot et al., 2016; Walton et al., 2017).

### DNA Methylation in Fatty Acid Metabolism Pathways Might Be a Marker for Persistent ADHD

We identified both DMRs and single DNA methylation sites in *APOB* and *LPAR5* to be differentially methylated between participants with persistent and remittent ADHD. Hypermethylation of *APOB* in participants with persistent ADHD compared to those with remittent ADHD implies lower *APOB* expression in those with persistent ADHD, but was not validated. *APOB* is the primary lipoprotein found on the surface of low density lipoproteins (LDL), which transports fatty acids and cholesterol throughout the body (Dashti et al., 2011). As indicated above, genes involved in fatty acid metabolism and fatty acid oxidation pathways have been indicated to be differentially methylated in patients with ADHD compared to healthy controls (Wilmot et al., 2016; Walton et al., 2017). This is also in concordance with previously published literature stating that lipid levels, specifically LDL levels, in blood are altered in patients with ADHD (Avcil, 2018; Pinho et al., 2018), and that LDL and APOB are the most discriminating lipid markers in children with ADHD (Irmisch et al., 2011). Moreover, the ADHD medication methylphenidate is known to alter blood lipid profiles (Charach et al., 2009), and a recent GWAS of ADHD found significant genetic correlations between ADHD and HDL cholesterol and LDL cholesterol (Demontis et al., 2019). A potential role of APOB in ADHD is underlined even more by the fact that mice knocked-out for the LDL-receptor (LDLR^−/−^) show hyperactivity (Elder et al., 2008). These mice lack the receptor for the LDL particles, containing APOB. However, it should be noted that lipoproteins functioning in the brain are exclusively produced in the brain, and therefore, blood and brain lipid levels might be very different from each other within individuals (Wang and Eckel, 2014). It was therefore important to find that blood DNA methylation levels for APOB correlated with brain DNA methylation levels in our analysis, suggesting similar expression regulation. APOB can be modified with oxidized aldehyde products, resulting in oxidized LDL (oxLDL) (Itabe et al., 2011), which can cause inflammation (Lara-Guzman et al., 2018). Another molecule that is produced by the oxidization process of LDL is LPA (Nsaibia et al., 2017). This molecule promotes inflammation after binding the LPAR5 (Ramesh et al., 2018). Mice depleted with LPA5 show nocturnal hyperactivity (Callaerts-Vegh et al., 2012), a second animal model in the APOB-related pathway showing ADHD symptoms. A more elaborate model of the potential role of APOB in persistent ADHD can be found in **Supplementary Figure 4** and the **Supplementary Text**.

### Strengths and Limitations

Our study shows several strengths and limitations. To our knowledge, we are the first to show differential methylation patterns in a cohort, in which we could compare participants with persistent and remittent ADHD. Although the sample size was small, patients and controls were carefully matched, and therefore, in terms of demographics, our group was as homogeneous as possible. Given the small sample size, it is promising that we find significant DMRs in two genes. However, all findings should be interpreted with caution, and results should be replicated in independent, bigger cohorts.

### Conclusion

We showed that hypermethylation of *APOB* and *LPAR5* in peripheral blood might be associated with persistence of ADHD, which is probably due to genetic variation influencing DNA methylation. These data, which suggest an involvement of fatty acid metabolism, provide new hypotheses for research into the molecular mechanism of persistent ADHD and associated traits.

## Data Availability Statement

The datasets for this article are not publicly available because of limitations in ethical approvals. A request procedure is in place, based on submission of a short proposal. Requests to access the datasets should be directed to BF, barbara.franke@radboudumc.nl or JB, jan.buitelaar@radboudumc.nl.

## Ethics Statement

The studies involving human participants were reviewed and approved by Centrale Commissie Mensgebonden Onderzoek: CMO Regio Arnhem Nijmegen; 2008/163; ABR: NL23894.091.08. Written informed consent to participate in this study was provided by the participants' legal guardian/next of kin.

## Author Contributions

Study conception and supervision: MM, MK, JM, BF. Obtained funding: CH, JO, DH, PH, JB, BF. Provided samples and/or data: CH, JO, DH, PH, JB. Conducted analyses and data interpretation: MM, MK, EH, DM, BF. Writing group: MM, MK, BF. All authors contributed to manuscript revision, read and approved the submitted version.

## Funding

This research was supported by an internal grant from the Donders Centre for Medical Neuroscience of Radboudumc. Support was also received from the Dutch National Science Agenda for the NWA NeurolabNL project (grant 400 17 602), and from the European Community's Horizon 2020 Programme (H2020/2014–2020) under grant agreement n° 728018 (Eat2beNICE). BF was also supported by a personal grant from the Netherlands Organization for Scientific Research (NWO) Vici Innovation Program (grant 016-130-669). The NeuroIMAGE project was supported by NIH Grant R01MH62873, NWO Large Investment Grant 1750102007010, and grants from Radboud University Medical Center, University Medical Center Groningen and Accare, and VU University Amsterdam. This work was also supported by grants from NWO Brain & Cognition (433-09-242 and 056-13-015) and from ZonMW (60-60600-97-193).

## Conflict of Interest

PH has received a research grant from and served on the advisory board for Shire. JB has served as a consultant, advisory board member, and/or speaker for Eli Lilly, JanssenCilag, Medice, Roche, Shire, and Servier. BF received educational speaking fees from Medice.

The remaining authors declare that the research was conducted in the absence of any commercial or financial relationships that could be construed as a potential conflict of interest.
