# Supplementary material for: Genome-Wide DNA Methylation Patterns in Persistent Attention-Deficit/Hyperactivity Disorder and in Association With Impulsive and Callous Traits
Source: Front Genet. 2020 Jan 31;11:16. doi: 10.3389/fgene.2020.00016 (PMC7005250; doi:10.3389/fgene.2020.00016)
Supplement: Supplementary file 1 [file DataSheet_1.docx]

**Supplementary Figure 1**
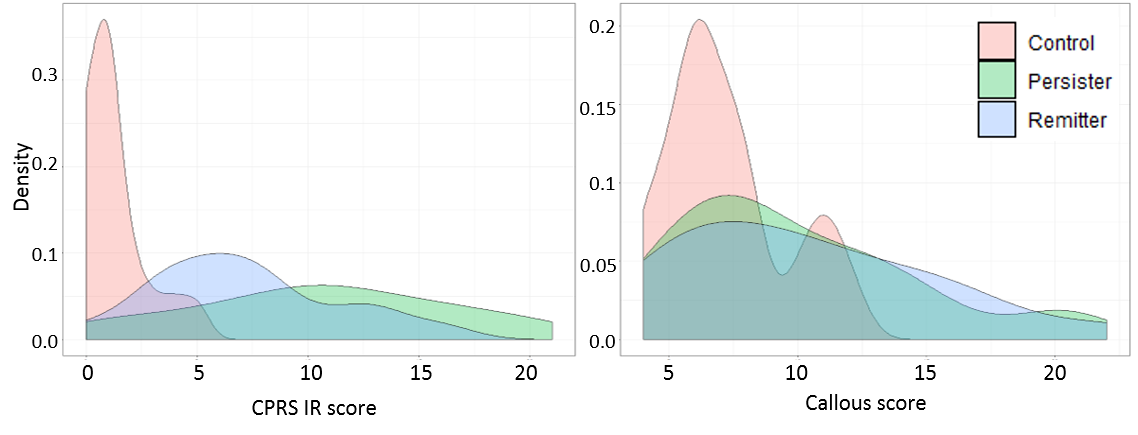
 **Impulsive and callous traits distributions in the NeuroIMAGE cohort.** Distributions of impulsive traits as measured by the Conners Parents Rating Scale – Impulsivity Restless domain (left) and callous traits as measured by the Inventory for Callous/Unemotional traits (right) were plotted for healthy controls (red), participants with persistent ADHD (green), and participants with remittent ADHD (blue).


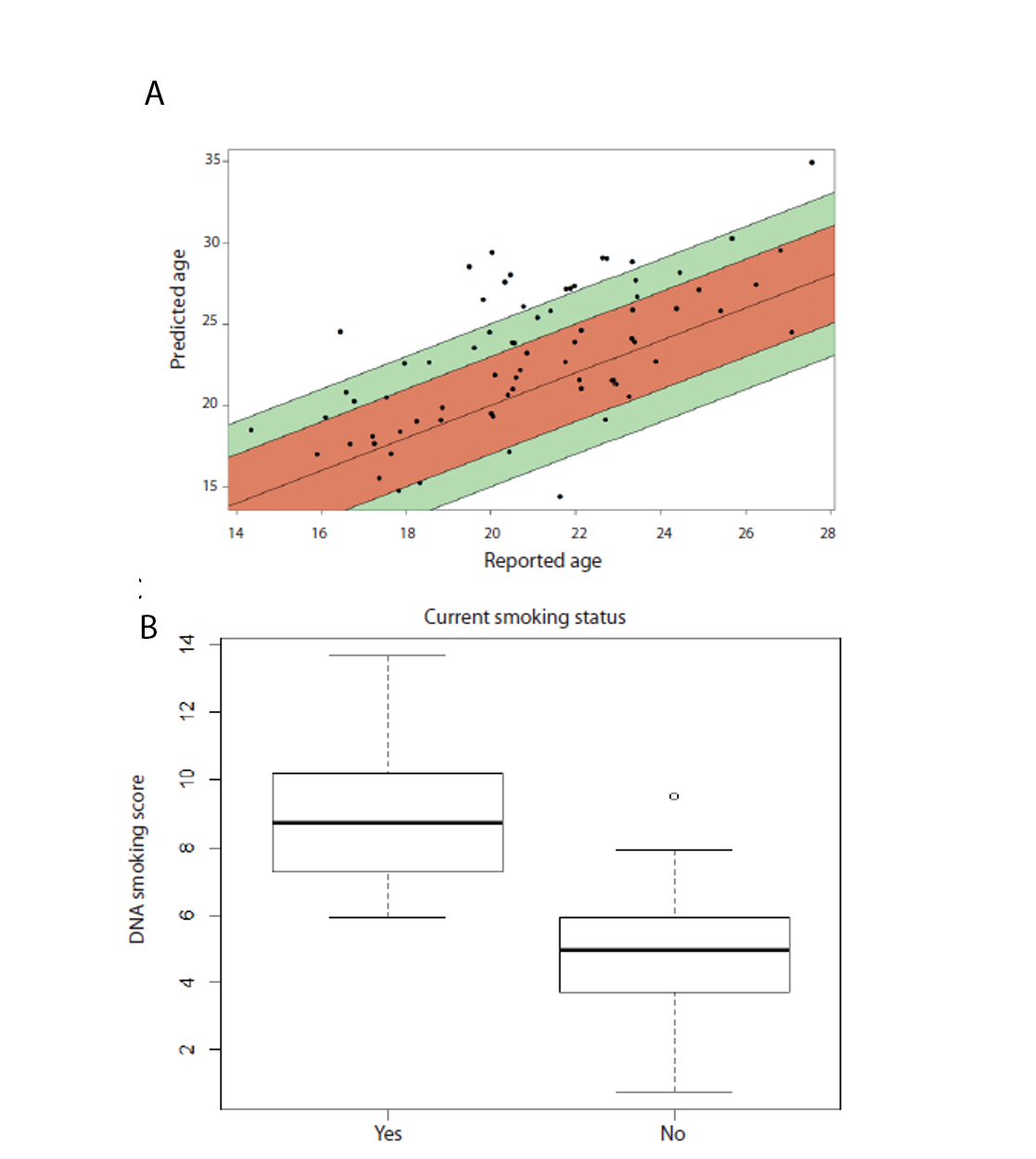


**Supplementary Figure 2 Quality control of samples and probes.** (A) Predicted age from Epigenetic Age Calculator against age at blood donation. Red area indicates samples whose estimate is within three years and green area within five years. For 51% of participants age was predicted within a range of 3 years around the actual age, and for almost 80% within an age range of 5 years. Individuals with a higher predicted than observed age are smokers. This may be explained by the fact that probes used to calculate smoking score are also included in predicting age. (B) Boxplot of smoking score derived from DNA methylation data of known smoking loci stratified by current smoking status.


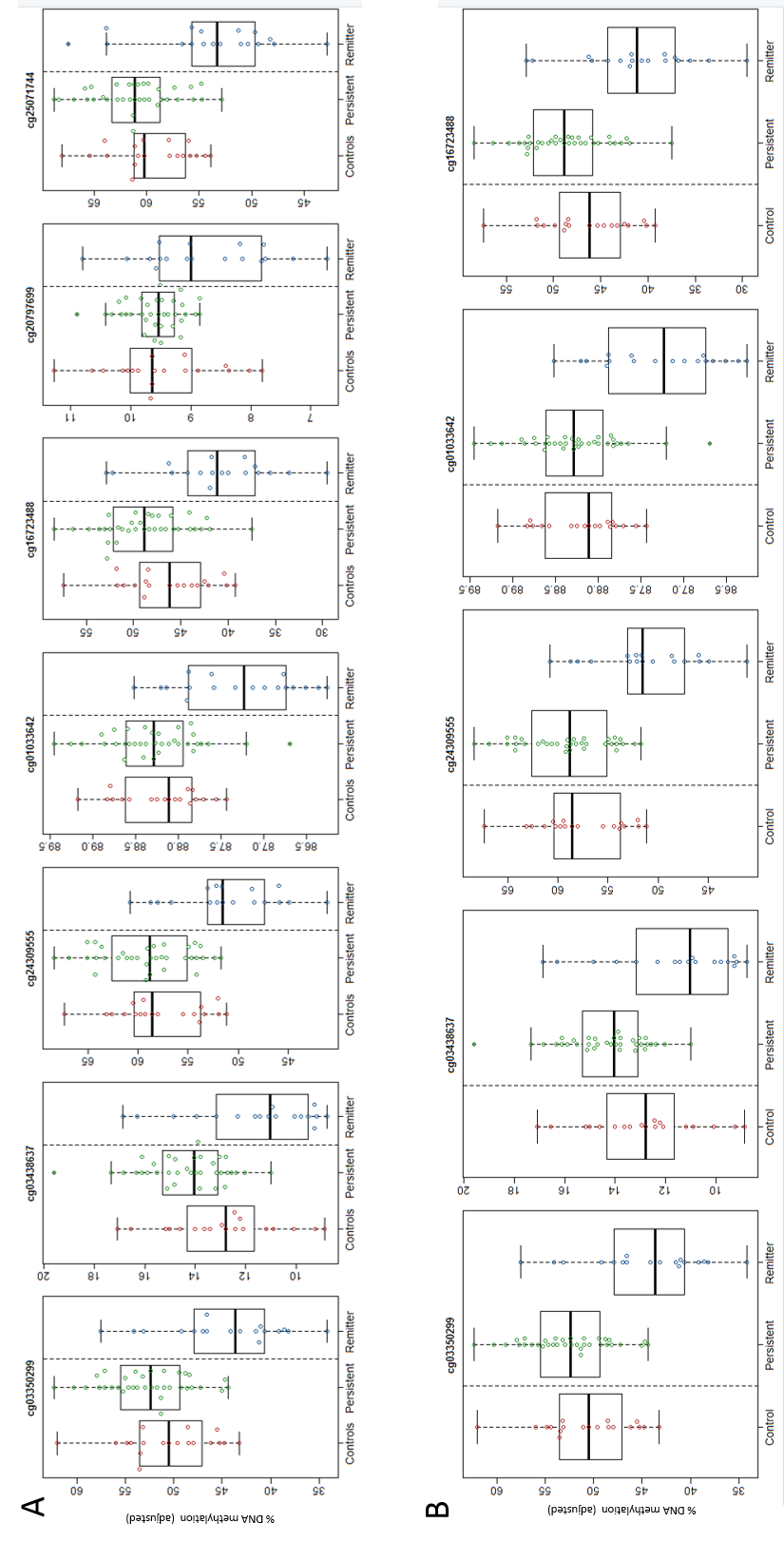


**Supplementary Figure 3 DNA methylation patterns of participants with remittent ADHD are distinct from participants with persistent ADHD and healthy controls.** Adjusted DNA methylation percentages of the nominally significant probes for the comparison between (A) participants with persistent ADHD (n=35) and healthy controls (n=19) and (B) participants with persistent and remittent ADHD (n=18) are plotted for every group.

**Supplementary Table 1. Baseline demographics of the included subcohort during the first wave (IMAGE 2003-2006)**

|  | **Healthy control** | **ADHD** | **Total**  **/ average** | **P-value (t-test)** |
| --- | --- | --- | --- | --- |
| **N** | 19 | 53 | 72 |  |
| **% male** | 68 | 67 | 73 | 0.67 |
| **Age**  **Mean (SD)** | 12.22 (2.77) | 11.45 (2.75) | 11.65 (2.75) | 0.30 |
| **IQ**  **Mean (SD)** | 110.5 (9.30) | 96.6 (13.01)  N=21 missing | 101.8 (13.51)  N=21 missing | 0.0002* |
| **Co-morbidities** | 0% Oppositional 0% Anxious 11% Probable ASD | 54% Oppositional 19% Anxious 86% Probable ASD (N=17 missing) | 40% Oppositional 14% Anxious 60% Probable ASD (N=17 missing) | <0.0001* 0.048* <0.0001* |
| **Medication status** | 0% N=1 missing | 73% N=1 missing | 53% N=2 missing | <0.0001* |
| **ADHD subtype** |  | 6% inattentive  94% combined |  |  |
| **Number of ADHD symptoms in parents** |  | 3.43 (3.95)  N=14 missing |  |  |

* significant p-value (<0.05) obtained by independent t-test
# Oppositional and anxious behavior defined by T score > 65 CPRS (mother), probable ASD (Autism Spectrum Disorder) defined as CSBQ population score > 6.

**Supplementary Table 2. Results for the suggestive significant methylation sites associated with persistent ADHD**

| CpG site | Genomic location | Gene | Location in gene | | | Effect size  (% difference) | P-value |
| --- | --- | --- | --- | --- | --- | --- | --- |
| *Persistent ADHD cases compared to healthy controls* | | | | | | | |
| cg19190762 | Chr13:44806055 | *SMIM2-AS1* | 5’UTR | | | -1.6 | 2.78*10^-6^ |
| cg02448002 | Chr9:1980116 | *JAK2* (>4970 bp) | Intergenic | | | -2.1 | 4.46*10^-6^ |
| cg18005896 | Chr6:168948135 | *SMOC2* | Intron 6 | | | 3.9 | 6.30*10^-6^ |
| cg10136166 | Chr8:144777909 | *BREA2* | TSS1500 | | | -1.0 | 6.91*10^-6^ |
| cg10811195 | Chr7:36433157 | *ANLN* | Intron 1 | | | 2.7 | 8.19*10^-6^ |
| cg19992207 | Chr2:111874495 | *ACOXL* | TSS1500 | | | 4.2 | 8.54*10^-6^ |
| cg24142353 | Chr7:15268685 | *AGMO* | Intron 12 | | | -2.3 | 8.70*10^-6^ |
| *Persistent compared to remittent ADHD cases* | | |  |  |  |  |  |
| cg03350299 | Chr2:21120465 | *APOB* | TSS200 | | | 9.8 | 3.48*10^-6^ |
| cg03438637 | Chr2:134292403 | *NCKAP5* | Intron 2 | | | 3.7 | 3.66*10^-6^ |
| cg24309555 | Chr2:21120461 | *APOB* | TS200 | | | 9.3 | 3.73*10^-6^ |
| cg01033642 | Chr16:80121693 | *RP11-525K10.1* | Non-coding | | | 1.1 | 5.04*10^-6^ |
| cg16723488 | Chr2:21120452 | *APOB* | TSS200 | | | 9.6 | 5.15*10^-6^ |

Significant methylation sites are shown for healthy controls (n = 19) and remittent ADHD cases (n = 18) compared to persistent ADHD cases (n = 35) with genomic location based on build 37, associated gene or nearest gene, location in gene, effect size (percentage methylation differences between remittent and persistent ADHD cases and healthy controls), and p-value.

TSS200 = within 200 bps upstream of the transcription start site

TSS1500 =within 1500 bps upstream of the transcription start site

**Supplementary Table 3 mQTLs of DMRs in APOB and LPAR5**

See corresponding excel file

**Supplementary Table 5 Predicted binding of transcription factors**

See corresponding excel file

**Supplementary Table 6 Blood-brain correlation of nominally significant DNA methylation sites**

See corresponding excel file

**Supplementary Table 7 Gene Ontology and KEGG pathway enrichment**

See corresponding excel file

**Supplementary Table 8 mQTLs for impulsivity and callousness**

See corresponding excel file

**Supplementary Table 4. Results for the suggestive significant methylation sites associated with impulsive and callous traits**

| CpG site | Genomic location | | Gene | Location in gene | Effect size  (% difference) | P-value |
| --- | --- | --- | --- | --- | --- | --- |
| *Impulsive traits* | | | | | | |
| Cg07402156 | Chr20:6054849 | | *FERMT1* (<643 bp) | Intergenic | -0.1 | 2.18*10^-7^ |
| Cg22157628 | Chr17:18547711 | | *TBC1D28* | 5’UTR | -0.1 | 5.88*10^-7^ |
| Cg22971038 | Chr6:39289004 | | *KCNK16* | Intron 1 | -0.1 | 2.12*10^-6^ |
| Cg02183491 | Chr13:99100829 | | *FARP1* | 3’UTR | -0.1 | 2.47*10^-6^ |
| Cg10654292 | Chr9:12526843 | | *MRRF* | TSS200 | -0.1 | 4.19*10^-6^ |
| Cg13894081 | Chr5:58335508 | | *PDE4D* | TSS200 | -0.1 | 4.21*10^-6^ |
| Cg20930398 | Chr6:31678458 | | *LY6G6E* | 3’UTR | -0.1 | 5.10*10^-6^ |
| Cg06908065 | Chr3:46318602 | | *CCR3* (<10405 bp) | Intergenic | 0.1 | 5.11*10^-6^ |
| Cg02595497 | Chr3:16850460 | | *PLCL2* | 5’UTR | 0.1 | 5.77*10^-6^ |
| Cg11457582 | Chr7:2107102 | | *MAD1L1* | 5’UTR | -0.1 | 6.15*10^-6^ |
| Cg01980298 | Chr19:17473410 | | *PLVAP* | Intron 3 | -0.1 | 7.44*10^-6^ |
| *Callous traits* | |  |  |  |  |  |
| Cg03758477 | Chr17:75210511 | | *SEC14L1* | 3’UTR | -0.2 | 2.17*10^-6^ |
| Cg19958417 | Chr14:104180744 | | *XRCC3* | 5’UTR | -0.2 | 2.33*10^-6^ |
| Cg14146711 | Chr22:38696843 | | *CSNK1E* | TSS1500 | 0.1 | 2.35*10^-6^ |
| Cg05007325 | Chr16:3474149 | | *ZNF597* | TSS1500 | 0.6 | 2.41*10^-6^ |
| Cg12365603 | Chr11:59052884 | | *RP11-1036E20.9* | TSS1500 | -0.2 | 7.95*10^-6^ |
| Cg02896594 | Chr9:81738435 | | *RP11-165H23.1* (<11902 bp) | Intergenic | -0.1 | 8.83*10^-6^ |

Significant methylation sites are shown for impulsive (n=71) and callous (n=70) traits with genomic location based on build 37, associated gene or nearest gene, location in gene, effect size (percentage methylation differences per point increase in trait score), and p-value.

TSS200 = within 200 bps upstream of the transcription start site

TSS1500 =within 1500 bps upstream of the transcription start site

**Supplementary text**

Epigenome-wide association studies (EWAS) for persistent ADHD and impulsive and callous traits indicated suggestive significant findings. Here, we report in depth on the findings that are not significant, but might harbour biological relevance signals.

## Biological interpretation of top-findings

To assess whether the observed levels of CpG methylation depend on genetic variation, methylation quantitative trait loci (meQTL) were assessed for all nominally significant CpG sites from the former analyses. Databases on meQTLs in peripheral blood during adolescence (1) and in the dorsal lateral prefrontal cortex in adults (2) were used. A SNP with <1 Mb distance to the corresponding CpG site was considered to be a trans-meQTL. In addition, expression quantitative trait methylations (eQTM) were assessed via the genome browser of iMETHYL, based on experiments in neutrophils isolated from 100 healthy Japanese subjects (3).

Since DNA methylation is known to be tissue-specific and can be used to distinguish neuronal from non-neuronal tissue (4), covariation patterns of DNA methylation in blood and the prefrontal cortex, entorhinal cortex, superior temporal gyrus, and cerebellum were obtained via the Blood Brain DNA Methylation Comparison Tool (5) for our nominally significant CpG sites. Moreover, we investigated gene expression profiles of the genes, in which nominally significant CpG sites were located, via Brainscope.nl (6), based on gene expression levels throughout the human brain as assessed by the Allen Human Brain Atlas (7). To predict whether methylation of the nominally significant CpG sites potentially influenced gene expression, we assessed whether any transcription factors (TFs) are known to bind to the identified differentially methylated CpG sites based on CHiP seq data of 119 TFs in the human embryonic stem cells TRANSFAC database (8), accessible via the USCS Genome Browser (9). With PROMO (10), TF binding sites were predicted in the forward string of 150 base pairs, including the CpG site of interest. Predicted TF binding sites were allowed to have a maximum dissimilarity rate of 15%. However, the methylated C always had to be predicted correctly to be considered a possible TF binding site. Correlations of expression patterns between the genes, in which a binding site was located, and the predicted TF were calculated throughout the whole brain. Genes and TFs with a Pearson correlation coefficient over 0.3 were considered to be hypothetically able to bind to each other.

The function ‘gometh’ of the R package ‘missMethyl’ was used to assess gene ontology (GO) term and KEGG pathway enrichment of genes annotated to relevant CpG sites (GRCh37 hg19). We included all sites with an association p<10^-3^ for one of our phenotypes, resulting in approximately 0.1% of all sites measured. To prevent bias, the hypergeometric test used takes into account that the number of CpG sites per gene on the EPIC array differs (11). Benjamini-Hochberg correction was used to account for multiple testing; we tested n=22,264 KEGG pathways and n=22,277 GO terms.

**2.1 DNA methylation profiles of healthy controls and participants with persistent and remittent ADHD differ**

While no individual probes passed the experiment-wide significance threshold in the comparison between healthy controls and patients with persistent ADHD, seven CpG sites showed differential methylation at our more lenient, suggestive significance threshold of p<5*10^-5^ (**Figure 1A, Supplementary Table 2, Supplementary Figure 3A**). Comparing individual DNA methylation differences between participants with persistent and remittent ADHD, we did not identify any differentially methylated probes passing the experiment-wide significance threshold (inflation factor=0.969). Five CpG sites showed differential methylation at our more lenient, suggestive significance threshold (p<5*10^-5^) (**Figure 1B, Supplementary Table 2, Supplementary Figure 3B**). Plots of the nominally significant CpG sites for all three groups can be found in **Supplementary Figure 3.**

**2.2 Differential methylation levels observed in DNA isolated from blood show correlation with brain methylation levels**

As DNA methylation can influence gene expression levels by making DNA less accessible for TFs, we aimed to predict, which TFs bind to the methylated cytosines in the selected CpG sites (using our more lenient, suggestive threshold). PAX5, TP53, NR3C1, and GCF were predicted to bind to multiple of these prioritized differentially methylated CpG sites (**Supplementary Table 5**). Since PAX5 has high co-expression with APOB throughout the whole brain in the AHBA data (r=0.30, p<2.2*10^-16^), it might be a plausible candidate TF for the regulation of *APOB*. Moreover, *NCKAP5* might be regulated by *GTF2I* (r=0.62, p<2.2*10^-16^) and *IRF1* (r=0.30, p<2.2*10^-16^).

To test, whether the differentially methylated CpG sites in persistent ADHD based on blood levels are also predictive for brain tissue DNA methylation, we reviewed the correlation between blood and brain DNA methylation. In general, correlation for our CpG sites of interest was variable (0.0041<r>0.77), but significant correlations between blood, prefrontal cortex, entorhinal cortex, superior temporal gyrus, and cerebellum were observed for all three CpG sites located in *APOB* (r>0.251, p<0.0301) as well as single probes in *SMOC2* (r>0.654, p<2*10-10), *BREA* (r>0.455, p<6.75*10-5)*,* and *AXOCL* (r>0.24, p<0.0384) (**Supplementary Table 6**).

**2.3 DNA methylation patterns in ADHD might be associated with cellular organization**

No GO terms and KEGG pathways were significantly enriched in the most differentially methylated CpG sites associated to ADHD persistence in both, the comparison with healthy controls and with participants with remittent ADHD after correction for multiple-testing. However, the most significant GO terms and KEGG pathways for the top genes observed in the comparison of participants with persistent ADHD and healthy controls were all involved in the regulation of actin organization (unadjusted p-value=3.5*10^-4^; **Supplementary Table 7**). Also for the CpG sites differentially methylated in the comparison between participants with remittent and persistent ADHD, the related GO term ‘actin filament base processes’ was among the two most enriched terms (unadjusted p-value=9.9*10^-5^), together with ribosomal function (unadjusted p-value=6.5*10^-5^; **Supplementary Table 7**); the most enriched KEGG pathways were PPAR signaling, pancreatic secretion, and aldosterone synthesis and secretion (unadjusted p-values=0.0015; **Supplementary Table 7**). None of the GO terms or KEGG pathways included *APOB* or *LPAR5* as key element.

## 2.4 DNA methylation profiles associated with impulsive and callous traits

Twelve CpG sites showed methylation patterns that correlated with impulsive traits (analyzed in n=70), of which the top hit was located 643 bp upstream of *FERMT1* (1% decreased methylation per unit increase in impulsivity score, p-value=2.18*10^-7^) (**Figure 2A, Supplementary Table 4**) (inflation factor=0.998). Another six CpG sites correlated with callousness (analyzed in n=71), of which the top hit was located in the 3’UTR of *SEC14L1* (2% decreased methylation per unit increase in callousness score, p-value=2.17*10^-6^) (**Figure 2B, Supplementary Table 4**) (inflation factor=0.980).

Of all the methylation sites associated with impulsive and callous traits according to our more lenient, suggestive significance threshold only the one in the 3’UTR of *FARP1* was a meQTL, suggesting a genomic effect on methylation (rs150430584, p-value=5.37*10^-9^; rs11640522, p-value=6.606*10^-8^; **Supplementary Table 8**).

## 2.5 DNA methylation profiles associated to callous traits might reflect brain DNA methylation

Assessing whether DNA methylation in peripheral blood correlated with that in brain, we found none of the methylation sites associated with impulsive traits to be significantly correlated with brain DNA methylation. Of the CpG sites for which methylation was associated with callousness, only those in *SEC14L1* and nearby *ZNF597* were captured on the array enabling the blood-brain correlation analysis; the correlation between blood and brain methylation for these probes was significant for the prefrontal cortex, entorhinal cortex, superior temporal gyrus, and cerebellum (Pearson’s r for blood – brain area comparison ranged from -0.17 to 0.77; p-value for blood – brain area comparison ranged from 0.99-2.45*10^-15^; **Supplementary Table 6**). TFs predicted to bind to motifs including the cytosines in CpG context were highly co-expressed with the genes containing nominally significant methylation sites associated with impulsivity or callousness (**Supplementary Table 5**). Among others, TFAP2A and GTF2I were predicted to bind motifs including the cytosine in *SEC14L1*.

After correction for multiple testing, no significant enrichment in GO terms or KEGG pathways was found in our findings for either impulsive or callous traits (**Supplementary Table 7**). For impulsivity, top findings were for the GO terms ‘memory’ (unadjusted p-value=2.7*10^-4^), ‘cell surface receptor signaling’ (unadjusted p-value=1.3*10^-3^), ‘adenylate cyclase activity’ (unadjusted p-value=1.3*10^-4^), and ‘L-ascorbic acid pathways’ (unadjusted p-value=1.4*10^-4^). Suggestive KEGG pathway enrichment for impulsive behavior was seen for ‘alpha-linoleic acid metabolism’ (unadjusted p-value=0.004) and ‘circadian entrainment’ (unadjusted p-value=0.0087). Uncorrected analyses for callousness hinted towards the enrichment of the GO term ‘cellular oxidative stress response’ (unadjusted p-value=0.00051) and the KEGG apoptosis pathway (unadjusted p-value=0.006).

**3.1 Blood DNA methylation associated with ADHD persistence correlated with brain DNA methylation**

We expected to find biomarkers of ADHD persistence and associated traits in the DNA isolated from peripheral blood. Such biomarkers do not necessarily reflect brain DNA methylation status, and we have no information on any causal relationships with disease. Some of our findings did show a relatively high correlation with methylation levels in different brain regions; especially the genes annotated to the differentially methylated CpG sites associated with callous traits are known to be highly expressed in the frontal lobe, striatum, amygdala, and hippocampus. The potential relevance of such findings is also supported by brain imaging studies of aggression, which showed that subcortical structures, particularly the amygdala and hippocampus, are involved in mediating aggressive behavior (12). The dorsolateral prefrontal cortex (DLPFC) and orbitofrontal cortex (OFC) receive input of the amygdala and have shown association with aggressive behavior as well (13). Moreover, the basal ganglia are targeted in treatments ameliorating impulsive aggressive behavior (14).

The lack of blood-brain correlation in methylation observed for many of our findings is not necessarily due to the limited sample size of our study. A recent methylome-wide association study for schizophrenia (N=1448) found that most of their top-findings were also specific for blood, but that half of their sites were located in genes that were enriched in brain-specific GO-terms (15). These findings suggest that the DNA methylation markers we identified in blood could also be relevant for brain-related processes and behaviors.

Based on genetic, rather than epigenetic studies, we did find support for the observed differential methylation sites associated with ADHD and impulsive and callous traits. Variants in *NCKAP5* and *AXOCL* have been found to be associated with inattention, before (16). Pde4d deregulation has been shown to cause inattentive and hyperactive symptoms in a mouse model (17). Lastly, genetic variants in *MAD1L1* have been found to be nominally associated with autism spectrum disorder, ADHD, bipolar disease, major depressive disorder, schizophrenia, and impulsive aggression in previous studies (18, 19). We also found some overlap in GO terms assigned to genes annotated to our single CpG sites and those found enriched in other studies, for example, van Dongen and co-workers as well as Wilmot and co-workers both identified DNA methylation profiles in genes involved in acetylcholine signaling to be associated to ADHD (symptoms) (20, 21), and we also identified nominally hypermethylated sites in acetylcholine signaling (*SEC14L1*).

**3.2 Hypermethylation of regions in *APOB* and *LPAR5* is associated with persistent ADHD pointing towards lipid metabolism as underlying biological mechanism**

In the regional analyses, we identified two hypermethylated regions in *APOB* and *LPAR5* when comparing persistent with remittent ADHD cases. *APOB* is the primary lipoprotein found on the surface of low density lipoproteins (LDL), which transports cholesterol throughout the body (22). Cholesterol is an important lipid in the maintenance of the brain and after binding to the LDL receptor (LDLR) it promotes axonal growth and neuronal survival (23, 24). The hypermethylation of *APOB* implies lower *APOB* expression in persistent ADHD cases compared to remittent ADHD cases, possibly by the regulation via the TFs PAX5 and TP53, which were both predicted to bind to the motif containing the cytosine in the CpG context of the gene. This is in concordance with previously published literature stating that lipid levels, specifically LDL levels, in blood are altered in patients with ADHD (25, 26) and LDL and APOB are the most discriminating lipid markers in children with ADHD (27). Moreover, the ADHD medication methylphenidate is also known to alter blood lipid profiles (28) and a recent GWAS found genetic correlations between lipid profiles and ADHD (29). The role of APOB in ADHD is underlined even more in LDLR -/- mice that show hyperactivity (30). However, it should be noted that lipoproteins functioning in the brain are exclusively produced in the brain and therefore, blood and brain lipid levels might be very different from each other within individuals (31), although blood DNA methylation levels for APOB correlated with brain DNA methylation levels in our analysis.


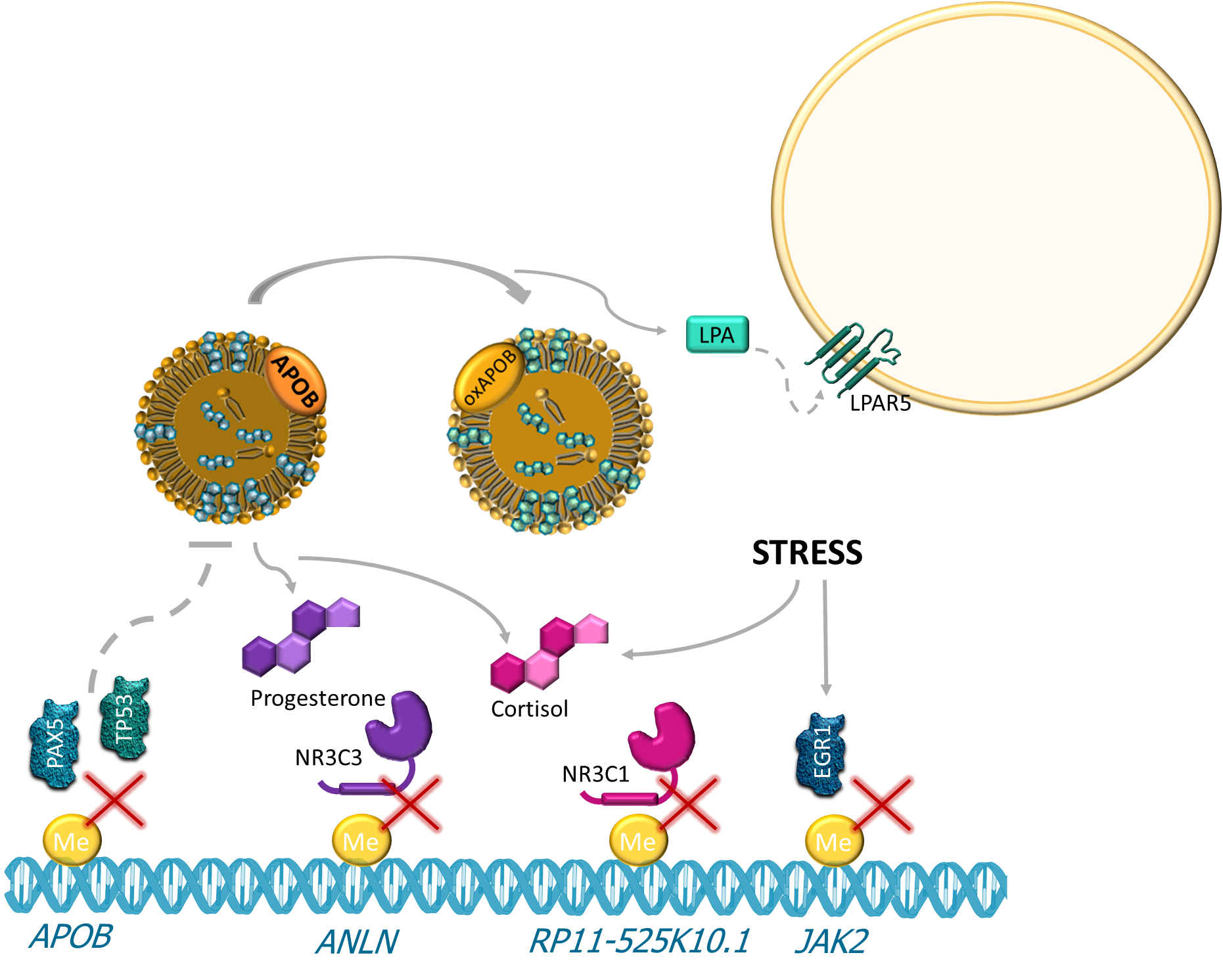


***Supplementary Figure 4*** ***Hypothesized mechanisms of APOB signalling in persistent ADHD.*** *Region of APOB is hypermethylated in persistent ADHD, resulting in decreased binding of the transcription factors PAX5 and/or TP53, causing less lipoproteins carrying cholesterol through the body. Cholesterol is the precursor of progesterone and cortisol, which will not be able to bind to their cognate receptors which are predicted to bind to the associated CpG sites in ANLN and RP11-525K10.1. Cortisol is released upon a stressor, as well EGR1, which is a predicted binding transcription factor at the CpG site in JAK2. Lastly, APOB can be oxidized, resulting in among others, LPA production. LPA can bind to LPAR5, a hypermethylated site in persistent ADHD.*

APOB can be modified with oxidized aldehyde products, resulting in oxidized LDL (oxLDL) (32), causing inflammation (33). Another product that is produced by the oxidization process of LDL is LPA (34). This molecule also promotes inflammation after binding the LPAR5 (35) and *Lpar5-/-* mice show nocturnal hyperactivity (36).

Cholesterol, transported by APOB-rich particles, is the precursor of various steroid hormones, such as progesterone and cortisol. Interestingly, the nuclear receptor NR3C3 has been predicted to bind to the motif containing the CpG site in *ANLN,* which was suggestively differentially methylated in persistent ADHD cases compared to healthy controls. NR3C3 is localized to the DNA after binding of progesterone and this substrate is thought to increase ADHD symptoms in rats (37) and humans (38). The increase of ADHD symptoms could thus be explained by the regulation of *ANLN*. Another interesting putative TF binding site has been identified in *RP11-525K10.1*. Here, NR3C1, the cognate receptor of cortisol can bind. Variants in this receptor gene have been found to be associated to ADHD symptoms and brain volume, underpinning the importance of stress reactivity in neurodevelopmental disorders (39). Interestingly, a previous DNA methylation study located a differentially methylated region upstream of the transcriptional start site associated with post-traumatic stress disorder symptom reduction (40). Stress has also been shown to increase Egr-1 expression in the hippocampus of an ADHD rat model male and showed increased Egr-1 expression in general in the prefrontal cortex and striatum (41). Moreover, ADHD medication, methylphenidate, increased Egr-1 expression in adult rat cortex and striatum in a dose-dependent manner (42, 43), We predicted EGR1 to be a putative TF for a CpG site differentially methylated CpG sites in persistent ADHD cases compared to healthy controls located nearby *JAK2* (44). This all leads to a hypothetical molecular model in persistent ADHD in the adult brain, involving stress-induced regulation of lipid metabolites.

**References**

1. Gaunt TR, Shihab HA, Hemani G, Min JL, Woodward G, Lyttleton O, et al. Systematic identification of genetic influences on methylation across the human life course. *Genome Biol* (2016) 17:61. Epub 2016/04/03. doi: 10.1186/s13059-016-0926-z. PubMed PMID: 27036880; PubMed Central PMCID: PMCPMC4818469.

2. Ng B, White CC, Klein HU, Sieberts SK, McCabe C, Patrick E, et al. An xQTL map integrates the genetic architecture of the human brain's transcriptome and epigenome. *Nat Neurosci* (2017) 20(10):1418-26. Epub 2017/09/05. doi: 10.1038/nn.4632. PubMed PMID: 28869584; PubMed Central PMCID: PMCPMC5785926.

3. Hachiya T, Furukawa R, Shiwa Y, Ohmomo H, Ono K, Katsuoka F, et al. Genome-wide identification of inter-individually variable DNA methylation sites improves the efficacy of epigenetic association studies. *NPJ Genom Med* (2017) 2:11. Epub 2017/12/22. doi: 10.1038/s41525-017-0016-5. PubMed PMID: 29263827; PubMed Central PMCID: PMCPMC5677974.

4. Ghosh S, Yates AJ, Fruhwald MC, Miecznikowski JC, Plass C, Smiraglia D. Tissue specific DNA methylation of CpG islands in normal human adult somatic tissues distinguishes neural from non-neural tissues. *Epigenetics* (2010) 5(6):527-38. Epub 2010/05/28. PubMed PMID: 20505344; PubMed Central PMCID: PMCPMC3322498.

5. Hannon E, Lunnon K, Schalkwyk L, Mill J. Interindividual methylomic variation across blood, cortex, and cerebellum: implications for epigenetic studies of neurological and neuropsychiatric phenotypes. *Epigenetics* (2015) 10(11):1024-32. Epub 2015/10/13. doi: 10.1080/15592294.2015.1100786. PubMed PMID: 26457534; PubMed Central PMCID: PMCPMC4844197.

6. Huisman SMH, van Lew B, Mahfouz A, Pezzotti N, Hollt T, Michielsen L, et al. BrainScope: interactive visual exploration of the spatial and temporal human brain transcriptome. *Nucleic Acids Res* (2017) 45(10):e83. Epub 2017/01/31. doi: 10.1093/nar/gkx046. PubMed PMID: 28132031; PubMed Central PMCID: PMCPMC5449549.

7. Hawrylycz MJ, Lein ES, Guillozet-Bongaarts AL, Shen EH, Ng L, Miller JA, et al. An anatomically comprehensive atlas of the adult human brain transcriptome. *Nature* (2012) 489(7416):391-9. Epub 2012/09/22. doi: 10.1038/nature11405. PubMed PMID: 22996553; PubMed Central PMCID: PMCPMC4243026.

8. Wingender E, Schoeps T, Haubrock M, Krull M, Donitz J. TFClass: expanding the classification of human transcription factors to their mammalian orthologs. *Nucleic Acids Res* (2018) 46(D1):D343-D7. Epub 2017/11/01. doi: 10.1093/nar/gkx987. PubMed PMID: 29087517; PubMed Central PMCID: PMCPMC5753292.

9. Kent WJ, Sugnet CW, Furey TS, Roskin KM, Pringle TH, Zahler AM, et al. The human genome browser at UCSC. *Genome Res* (2002) 12(6):996-1006. Epub 2002/06/05. doi: 10.1101/gr.229102. PubMed PMID: 12045153; PubMed Central PMCID: PMCPMC186604.

10. Messeguer X, Escudero R, Farre D, Nunez O, Martinez J, Alba MM. PROMO: detection of known transcription regulatory elements using species-tailored searches. *Bioinformatics* (2002) 18(2):333-4. Epub 2002/02/16. PubMed PMID: 11847087.

11. Geeleher P, Hartnett L, Egan LJ, Golden A, Raja Ali RA, Seoighe C. Gene-set analysis is severely biased when applied to genome-wide methylation data. *Bioinformatics* (2013) 29(15):1851-7. Epub 2013/06/05. doi: 10.1093/bioinformatics/btt311. PubMed PMID: 23732277.

12. Rosell DR, Siever LJ. The neurobiology of aggression and violence. *CNS Spectr* (2015) 20(3):254-79. Epub 2015/05/06. doi: 10.1017/S109285291500019X. PubMed PMID: 25936249.

13. Lane SD, Kjome KL, Moeller FG. Neuropsychiatry of aggression. *Neurol Clin* (2011) 29(1):49-64, vii. Epub 2010/12/22. doi: 10.1016/j.ncl.2010.10.006. PubMed PMID: 21172570; PubMed Central PMCID: PMCPMC3053027.

14. Sugden SG, Kile SJ, Hendren RL. Neurodevelopmental pathways to aggression: a model to understand and target treatment in youth. *J Neuropsychiatry Clin Neurosci* (2006) 18(3):302-17. Epub 2006/09/12. doi: 10.1176/jnp.2006.18.3.302. PubMed PMID: 16963580.

15. Chan RF, Shabalin AA, Montano C, Hannon E, Hultman CM, Fallin MD, et al. Independent Methylome-Wide Association Studies of Schizophrenia Detect Consistent Case-Control Differences. *Schizophr Bull* (2019). Epub 2019/06/06. doi: 10.1093/schbul/sbz056. PubMed PMID: 31165892.

16. Lasky-Su J, Neale BM, Franke B, Anney RJ, Zhou K, Maller JB, et al. Genome-wide association scan of quantitative traits for attention deficit hyperactivity disorder identifies novel associations and confirms candidate gene associations. *Am J Med Genet B Neuropsychiatr Genet* (2008) 147B(8):1345-54. Epub 2008/09/30. doi: 10.1002/ajmg.b.30867. PubMed PMID: 18821565.

17. D'Andrea I, Fardella V, Fardella S, Pallante F, Ghigo A, Iacobucci R, et al. Lack of kinase-independent activity of PI3Kgamma in locus coeruleus induces ADHD symptoms through increased CREB signaling. *EMBO Mol Med* (2015) 7(7):904-17. Epub 2015/04/18. doi: 10.15252/emmm.201404697. PubMed PMID: 25882071; PubMed Central PMCID: PMCPMC4520656.

18. Cross-Disorder Group of the Psychiatric Genomics C. Identification of risk loci with shared effects on five major psychiatric disorders: a genome-wide analysis. *Lancet* (2013) 381(9875):1371-9. Epub 2013/03/05. doi: 10.1016/S0140-6736(12)62129-1. PubMed PMID: 23453885; PubMed Central PMCID: PMCPMC3714010.

19. Montalvo-Ortiz JL, Zhang H, Chen C, Liu C, Coccaro EF. Genome-Wide DNA Methylation Changes Associated with Intermittent Explosive Disorder: A Gene-Based Functional Enrichment Analysis. *Int J Neuropsychopharmacol* (2018) 21(1):12-20. Epub 2017/11/07. doi: 10.1093/ijnp/pyx087. PubMed PMID: 29106553; PubMed Central PMCID: PMCPMC5789263.

20. van Dongen J, Zilhao NR, Sugden K, Consortium B, Hannon EJ, Mill J, et al. Epigenome-wide Association Study of Attention-Deficit/Hyperactivity Disorder Symptoms in Adults. *Biol Psychiatry* (2019). Epub 2019/04/21. doi: 10.1016/j.biopsych.2019.02.016. PubMed PMID: 31003786.

21. Wilmot B, Fry R, Smeester L, Musser ED, Mill J, Nigg JT. Methylomic analysis of salivary DNA in childhood ADHD identifies altered DNA methylation in VIPR2. *J Child Psychol Psychiatry* (2016) 57(2):152-60. Epub 2015/08/26. doi: 10.1111/jcpp.12457. PubMed PMID: 26304033; PubMed Central PMCID: PMCPMC4724325.

22. Dashti M, Kulik W, Hoek F, Veerman EC, Peppelenbosch MP, Rezaee F. A phospholipidomic analysis of all defined human plasma lipoproteins. *Sci Rep* (2011) 1:139. Epub 2012/02/23. doi: 10.1038/srep00139. PubMed PMID: 22355656; PubMed Central PMCID: PMCPMC3216620.

23. Ladu MJ, Reardon C, Van Eldik L, Fagan AM, Bu G, Holtzman D, et al. Lipoproteins in the central nervous system. *Ann N Y Acad Sci* (2000) 903:167-75. Epub 2000/05/20. PubMed PMID: 10818504.

24. Hayashi H, Campenot RB, Vance DE, Vance JE. Apolipoprotein E-containing lipoproteins protect neurons from apoptosis via a signaling pathway involving low-density lipoprotein receptor-related protein-1. *J Neurosci* (2007) 27(8):1933-41. Epub 2007/02/23. doi: 10.1523/JNEUROSCI.5471-06.2007. PubMed PMID: 17314289.

25. Avcil S. Association between altered lipid profiles and attention deficit hyperactivity disorder in boys. *Nord J Psychiatry* (2018) 72(5):361-6. Epub 2018/04/25. doi: 10.1080/08039488.2018.1465591. PubMed PMID: 29688116.

26. Pinho R, Wang B, Becker A, Rothenberger A, Outeiro TF, Herrmann-Lingen C, et al. Attention-deficit/hyperactivity disorder is associated with reduced levels of serum low-density lipoprotein cholesterol in adolescents. Data from the population-based German KiGGS study. *World J Biol Psychiatry* (2018):1-9. Epub 2017/12/16. doi: 10.1080/15622975.2017.1417636. PubMed PMID: 29243550.

27. Irmisch G, Thome J, Reis O, Hassler F, Weirich S. Modified magnesium and lipoproteins in children with attention deficit hyperactivity disorder (ADHD). *World J Biol Psychiatry* (2011) 12 Suppl 1:63-5. Epub 2011/08/05. doi: 10.3109/15622975.2011.600292. PubMed PMID: 21812620.

28. Charach G, Kaysar N, Grosskopf I, Rabinovich A, Weintraub M. Methylphenidate has positive hypocholesterolemic and hypotriglyceridemic effects: new data. *J Clin Pharmacol* (2009) 49(7):848-51. Epub 2009/06/26. doi: 10.1177/0091270009336736. PubMed PMID: 19553406.

29. Demontis D, Walters RK, Martin J, Mattheisen M, Als TD, Agerbo E, et al. Discovery of the first genome-wide significant risk loci for attention deficit/hyperactivity disorder. *Nat Genet* (2019) 51(1):63-75. Epub 2018/11/28. doi: 10.1038/s41588-018-0269-7. PubMed PMID: 30478444; PubMed Central PMCID: PMCPMC6481311.

30. Elder GA, Ragnauth A, Dorr N, Franciosi S, Schmeidler J, Haroutunian V, et al. Increased locomotor activity in mice lacking the low-density lipoprotein receptor. *Behav Brain Res* (2008) 191(2):256-65. Epub 2008/05/10. doi: 10.1016/j.bbr.2008.03.036. PubMed PMID: 18466986; PubMed Central PMCID: PMCPMC4662864.

31. Wang H, Eckel RH. What are lipoproteins doing in the brain? *Trends Endocrinol Metab* (2014) 25(1):8-14. Epub 2013/11/06. doi: 10.1016/j.tem.2013.10.003. PubMed PMID: 24189266; PubMed Central PMCID: PMCPMC4062975.

32. Itabe H, Obama T, Kato R. The Dynamics of Oxidized LDL during Atherogenesis. *J Lipids* (2011) 2011:418313. Epub 2011/06/11. doi: 10.1155/2011/418313. PubMed PMID: 21660303; PubMed Central PMCID: PMCPMC3108093.

33. Lara-Guzman OJ, Gil-Izquierdo A, Medina S, Osorio E, Alvarez-Quintero R, Zuluaga N, et al. Oxidized LDL triggers changes in oxidative stress and inflammatory biomarkers in human macrophages. *Redox Biol* (2018) 15:1-11. Epub 2017/12/02. doi: 10.1016/j.redox.2017.11.017. PubMed PMID: 29195136; PubMed Central PMCID: PMCPMC5723280.

34. Nsaibia MJ, Boulanger MC, Bouchareb R, Mkannez G, Le Quang K, Hadji F, et al. OxLDL-derived lysophosphatidic acid promotes the progression of aortic valve stenosis through a LPAR1-RhoA-NF-kappaB pathway. *Cardiovasc Res* (2017) 113(11):1351-63. Epub 2017/05/05. doi: 10.1093/cvr/cvx089. PubMed PMID: 28472283; PubMed Central PMCID: PMCPMC5852522.

35. Ramesh S, Govindarajulu M, Suppiramaniam V, Moore T, Dhanasekaran M. Autotaxin(-)Lysophosphatidic Acid Signaling in Alzheimer's Disease. *Int J Mol Sci* (2018) 19(7). Epub 2018/06/24. doi: 10.3390/ijms19071827. PubMed PMID: 29933579; PubMed Central PMCID: PMCPMC6073975.

36. Callaerts-Vegh Z, Leo S, Vermaercke B, Meert T, D'Hooge R. LPA5 receptor plays a role in pain sensitivity, emotional exploration and reversal learning. *Genes Brain Behav* (2012) 11(8):1009-19. Epub 2012/10/09. doi: 10.1111/j.1601-183X.2012.00840.x. PubMed PMID: 23039190.

37. Kozlowska A, Wojtacha P, Rowniak M, Kolenkiewicz M, Tsai ML. Differences in serum steroid hormones concentrations in spontaneously hypertensive rats (SHR) - an animal model of attention-deficit/hyperactivity disorder (ADHD). *Physiol Res* (2018). Epub 2018/11/16. PubMed PMID: 30433797.

38. Roberts B, Eisenlohr-Moul T, Martel MM. Reproductive steroids and ADHD symptoms across the menstrual cycle. *Psychoneuroendocrinology* (2018) 88:105-14. Epub 2017/12/05. doi: 10.1016/j.psyneuen.2017.11.015. PubMed PMID: 29197795; PubMed Central PMCID: PMCPMC5803442.

39. van der Meer D, Hoekstra PJ, Bralten J, van Donkelaar M, Heslenfeld DJ, Oosterlaan J, et al. Interplay between stress response genes associated with attention-deficit hyperactivity disorder and brain volume. *Genes Brain Behav* (2016) 15(7):627-36. Epub 2016/07/09. doi: 10.1111/gbb.12307. PubMed PMID: 27391809.

40. Vinkers CH, Geuze E, van Rooij SJH, Kennis M, Schur RR, Nispeling DM, et al. Successful treatment of post-traumatic stress disorder reverses DNA methylation marks. *Mol Psychiatry* (2019). Epub 2019/10/28. doi: 10.1038/s41380-019-0549-3. PubMed PMID: 31645664.

41. Clements KM, Wainwright PE. Swim stress increases hippocampal Zif268 expression in the spontaneously hypertensive rat. *Brain Res Bull* (2010) 82(5-6):259-63. Epub 2010/05/12. doi: 10.1016/j.brainresbull.2010.05.002. PubMed PMID: 20457228.

42. Van Waes V, Beverley J, Marinelli M, Steiner H. Selective serotonin reuptake inhibitor antidepressants potentiate methylphenidate (Ritalin)-induced gene regulation in the adolescent striatum. *Eur J Neurosci* (2010) 32(3):435-47. Epub 2010/08/14. doi: 10.1111/j.1460-9568.2010.07294.x. PubMed PMID: 20704593; PubMed Central PMCID: PMCPMC2921647.

43. Yano M, Steiner H. Methylphenidate (Ritalin) induces Homer 1a and zif 268 expression in specific corticostriatal circuits. *Neuroscience* (2005) 132(3):855-65. Epub 2005/04/20. doi: 10.1016/j.neuroscience.2004.12.019. PubMed PMID: 15837145.

44. Wang J, Zhuang J, Iyer S, Lin X, Whitfield TW, Greven MC, et al. Sequence features and chromatin structure around the genomic regions bound by 119 human transcription factors. *Genome Res* (2012) 22(9):1798-812. Epub 2012/09/08. doi: 10.1101/gr.139105.112. PubMed PMID: 22955990; PubMed Central PMCID: PMCPMC3431495.
